# Systemic lupus erythematosus and Raynaud's phenomenon*

**DOI:** 10.1590/abd1806-4841.20153881

**Published:** 2015

**Authors:** Flavia Emilie Heimovski, Juliana A. Simioni, Thelma Larocca Skare

**Affiliations:** 1 Faculdade Evangélica do Paraná (Fepar) - Curitiba (PR), Brazil

**Keywords:** Autoantibodies, Lupus erythematosus, systemic, Prognosis, Raynaud disease

## Abstract

**BACKGROUND:**

Patients with systemic lupus erythematosus seem to belong to different serological and clinical subgroups of the disease. Genetic background can cause the appearance of these subgroups.

**OBJECTIVE:**

To determine whether Brazilian patients who have systemic lupus erythematosus and Raynaud's phenomenon differ from those who do not.

**METHODS:**

Retrospective analysis of 373 medical records of systemic lupus erythematosus patients studied for demographic, clinical and serological data. A comparative analysis was performed of individuals with and without RP.

**RESULTS:**

There was a positive association between Raynaud's phenomenon and age at diagnosis (p=0.02), presence of anti-Sm (p=0.01) antibodies and anti-RNP (p<0.0001). Furthermore, a negative association was found between Raynaud's phenomenon and hemolysis (p=0.01), serositis (p=0.01), glomerulonephritis (p=0.0004) and IgM aCL (p=0.004) antibodies.

**CONCLUSION:**

Raynaud's phenomenon patients appear to belong to a systemic lupus erythematosus subset with a spectrum of clinical manifestations located in a more benign pole of the disease.

## INTRODUCTION

Raynaud's phenomenon (RP) is a nonspecific cutaneous lesion that appears in 18-46 % of patients with systemic lupus erythematosus (SLE).^1,2^ It results from a vasospasm triggered by cold conditions or emotional stress that causes blanching, cyanosis, and reactive hyperemia of extremities.^1^ RP is caused by vasoconstriction of the digital arteries, precapillary arterioles and cutaneous arteriovenous shunts; it has also been associated with digital ischemia and ulcers.^3,4^

SLE is an illness in which genetic background affects not only the disease's prevalence but also its phenotype.^5^ This allows for the appearance of clusters of autoantibodies and clinical findings that define the disease's subtypes.^6,7^ Knowledge of these clusters enables clinicians treating patients with given symptoms to watch out for those that are associated with it.

Some authors have linked the presence of RP to pulmonary hypertension; others have associated it with nervous system involvement.^1,8^ However, it remains unknown if the presence of RP in SLE patients suggests a different course of the disease in Brazilian patients.

This study analyzed the prevalence of RP in a sample of Brazilian SLE patients and whether this finding is associated with a peculiar clinical and serological profile.

## METHODS

This retrospective study reviewed 373 charts from a single tertiary center, relating to SLE patients seen in the last 10 years, and approved by the local Research Ethics Committee. To be included patients must fulfill at least four of the 1997 revised American College of Rheumatology classification criteria for systemic lupus erythematosus.^9^ The study excluded patients diagnosed with the disease before the age of 16 and those with incomplete records. Data on demographic, clinical and serological profiles were obtained. The analyzed data refer to a non-probabilistic sample, with sequential and intentional selection, respecting the inclusion and exclusion criteria. The definition of clinical findings was that adopted in the ACR classification criteria.^9^ Patients were divided into two groups: those with and those without RP; they were then compared.

All data obtained were collected in frequency and contingency tables. The Kolmogorov-Sminorv test was used to study data distribution. Central tendency was expressed in median and interquartile range (IQR) as all numeric data were non-parametrical. Association studies were performed via Fisher's and chi-squared tests for nominal data, and through the Mann Whitney test for numerical data. Calculations were carried out with the help of the Graph Pad prism version 5.0 software. The significance adopted was of 5%.

## RESULTS

The studied sample had a 66.1% prevalence of auto-declared Caucasians and a 33.9% prevalence of auto-declared Afrodescendants, with a median disease duration of 48 months (range 1-384 months; IQR =12-72) and a median diagnosis age of 31 years (range 16-73 years; IQR=23-40). In this sample, 93.8% of patients were females, while 6.2% of patients were males.

The main clinical and serological findings are displayed in table 1.

**Table 1:** Clinical and serological profile of 373 systemic lupus erythematosus patients

	N	%
Discoid lesion	48/355	14%
Butterfly rash	195/351	55.5%
Photosensitivity	263/367	71.6%
Oral ulcers	160/359	44.6%
Arthritis	222/371	59.8%
Serositis	47/218	21.5%
Hemolysis	29/364	7.9%
Leukopenia	98/359	27.3%
Thrombocytopenia	86/353	24%
Glomerulonephritis	163/367	44.4%
Seizures	38/365	10.4%
Psycosis	15/215	6.9%
Anti-Ro	128/345	37.1%
Anti-La	66/345	19.1%
Anti-Sm	81/340	23.8%
Anti-dsDNA	124/350	35.8%
Anti- RNP	83/266	31.2%
Anticardiolipin IgG	48/349	13.7%
Anticardiolipin IgM	37/346	10.6%
Lupus anticoagulant	39/281	13.9%
Rheumatoid factor	88/322	27.3%

In this sample, the prevalence of Raynaud's phenomenon was of 183/373 or 49.1%.

Comparing lupus patients with and without RP, we found data in table 2 showing that RP was more common in older patients and in those with anti-RNP and anti-Sm. Glomerulonephritis, serositis, hemolytic anemia and anticardiolipin IgM antibodies were less common in this group.

**Table 2:** Association studies with Raynaud's phenomenon (RP) in 373 systemic lupus erythematosus patients

	RP positive	RP negative	P
	N=183	N=190	
Gender (male/female)	11/172	12/178	0.90
Disease duration (months)	1 to 384	12 to 372	0.49
	Median 48 (IQR=12-72)	Median 36.0 (IQR=12-72)	
Age at diagnosis (years)	16-73	16-64	0.02
	Median 33 (IQR=24-42)	Median 29.5 (IQR=22-38)	
Tobacco exposure	60/160 - 37.5%	60/173 -34.6%	0.59
Discoid lesion	28/173 - 16.2%	20/182 - 10.9%	0.15
Butterfly rash	99/173 - 57.2%	96/178 - 53.6%	0.53
Photosensitivity	134/179 - 74.8%	129/188 - 68.6%	0.18
Oral ulcers	80/174 - 45.9%	80/185 - 43.2%	0.60
Arthritis	105/183 - 57.3%	117/188 - 62.2%	0.34
Serositis	14/98 - 14.2%	33/120 - 27.5%	0.01
Hemolitic anemia	8/176 - 4.5%	21/188 - 11.1%	0.01
Leukopenia	51/176 - 28.9%	47/183 - 25.7%	0.48
Thrombocytopenia	49/172 - 28.4%	37/181- 20.4%	0.07
Glomerulonephritis	65/ 183 - 35.5%	102/190 - 53.6%	0.0004
Seizures	13/176 - 7.3%	15/189 - 13.2%	0.06
Psychosis	8/98 - 8.1%	7/117 - 5.9%	0.53
Anti-Ro	68/169 - 40.2%	60/176 - 34.1%	0.23
Anti-La	37/169 - 21.8%	29/176 - 16.4%	0.20
Anti-Sm	49/167 - 29.3%	32/173 - 18.5%	0.01
Anti-dsDNA	59/168 - 35.1%	65/182 - 35.7%	0.90
Anti-RNP	55/126 - 43.6%	28/140 - 20%	<0.0001
Anticardiolpin IgG	19/170 - 11.1%	29/179 - 16.2%	0.17
Anticardiolpin IgG	10/170 - 5.8%	27/176 - 15.3%	0.004
Lupus anticoagulant	17/135 - 12.6%	22/146 - 15.1%	0.54
Rheumatoid factor	49/163 - 30.1%	39/159 - 24.5%	0.26

## DISCUSSION

Our results suggest that patients with RP experience disease onset at older ages and have less glomerulonephritis, which is one of the most serious manifestations of SLE.10 Approximately 10 to 30 % of patients with the proliferative form progress to endstage renal disease, needing dialysis or kidney transplants.^10^ In this context, the presence of RP would suggest a less severe disease. A study in 79 Serbian patients failed to demonstrate any link between RP and glomerulonephritis, while another with a larger number of American patients (n=1.357) confirmed the negative association we found.^1,4^

It is consistently observed that RF is more common in people who experience disease onset at more advanced ages and in whom the lupus is considered less severe.^4^

This finding is highly important for clinics that monitor patients because it allows inferences to be made about the disease's prognosis, by drawing on simple clinical data obtained via anamnesis, such as the presence of RF.

Furthermore, the established association of RP with anti-RNP and anti-Sm autoantibodies has been noted by other researchers.7 Anti-RNP and anti-Sm antibodies are directed against spliceosome proteins and provoke the appearance of an ANA with a speckled pattern.^11,12^ Spliceosome is an intracellular structure that removes the intronic sequences of the pre-messenger RNA and links protein coding sequences to form mature RNA.^11^ RNP presence is considered necessary for mixed connective tissue disease diagnosis, but it appears in almost all connective tissue diseases.^13^ Anti-Sm is one of the most specific autoantibodies in SLE and is detected in 5 to 30% of SLE patients. It is nearly always accompanied by anti-RNP antibodies.^13,14,15^ Interestingly, Tapanes et al. had already associated the Sm/RNP group with the less severe form or with the absence of SLE glomerulonephritis.^16^ In addition, Hoffman et al.^17^ reported that SLE patients with Sm/RNP antibodies had a lower prevalence of urine cellular casts, while To et al.^7^ observed that patients in the Sm/RNP cluster had the lowest incidence of renal manifestations.

The true value of the anti-RNP antibody is uncertain as it has not yet been linked to the disease's pathophysiological process. Some authors have regarded it merely as a disease marker.^13^ Interestingly, Levy et al.^18^ described the case of a lupus patient in which the appearance of severe RP was associated with the emergence of high titer anti-RNP seroconversion, suggesting that these autoantibodies may play a role in the pathogenesis of peripheral ischemic attacks.

Other negative clinical associations identified here and associated with hemolytic anemia and serositis, were not confirmed by Pavlov-Dolijanovic et al..^1^ This relationship may be peculiar to the local ethnic background.

Finally, anticardiolipin (aCl) antibodies have been studied in this context, yielding conflicting results.^19,20^ RP is caused by vasospasm of the small muscular arteries and arterioles of the digits, not by thrombosis.^19^ Nevertheless thrombotic events may complicate severe forms with sustained vasospasm; patients affected by recalcitrant disease may benefit from anticoagulant therapy.^9^ Vayssairat et al.^20^ found a significantly higher prevalence of aCL IgG in patients with RP and connective tissue disorders than in healthy controls. However, it is necessary to highlight that the prevalence of either antiphospholipid antibodies or RP is significantly higher in patients with connective tissue disorders than in the normal population. In a case-control study, limited to only 35 patients without SLE but with clinical disorders such as thrombosis, repeated abortions and thrombocytopenia, a positive association was found between RP and aCls.^21^ In contrast, a study of 93 SLE patients failed to establish any associations with this group of antibodies.^19^ We detected that aCl IgM was less common in patients with RP; other antiphospholipid antibodies did not show any association. ACl IgM is an antiphospholipid antibody that is less inclined to causing thrombosis than aCl IgG.^22^

## CONCLUSION

The present findings suggest that although RP is considered a non-specific cutaneous lesion, it does provide important information on associated symptoms and the autoantibody profile in SLE. Its presence allows the establishment of a disease subgroup in which patients experience disease onset at an older age, presenting with clinical peculiarities such as lower prevalence of glomerulonephritis, serositis and hemolytic anemia. These patients therefore have a better prognosis. Still, this subgroup of SLE patients with FR is serologically marked by the autoantibodies anti-RNP and anti-Sm.

Identifying the different patterns of SLE presentation is important from a prognostic point of view, as the recognition of a simple clinical finding such as FR allows the attending doctor to predict the disease's evolution.
